# Endophytic Bacterial Biofilm-Formers Associated with Antarctic Vascular Plants

**DOI:** 10.3390/microorganisms12101938

**Published:** 2024-09-25

**Authors:** Olga Iungin, Yevheniia Prekrasna-Kviatkovska, Oleksandr Kalinichenko, Olena Moshynets, Geert Potters, Marina Sidorenko, Yaroslav Savchuk, Saulius Mickevičius

**Affiliations:** 1 Department of Biotechnology, Leather and Fur, Faculty of Chemical and Biopharmaceutical Technologies, Kyiv National University of Technologies and Design, 01011 Kyiv, Ukraine; ktshh@knutd.edu.ua; 2 Biofilm Study Group, Department of Cell Regulatory Mechanisms, Institute of Molecular Biology and Genetics, National Academy of Sciences of Ukraine, 03143 Kyiv, Ukraine; moshynets@gmail.com; 3 Faculty of Natural Sciences, Vytautas Magnus University, 53361 Kaunas, Lithuania; marina.sidorenko@vdu.lt (M.S.); saulius.mickevicius@vdu.lt (S.M.); 4 Biology and Ecology Department, State Institution National Antarctic Scientific Center, 01601 Kyiv, Ukraine; preckrasna@gmail.com; 5 AMACORT, Nautical Faculty, Antwerp Maritime Academy, 2030 Antwerp, Belgium; geert.potters@hzs.be; 6 Department of Bioscience Engineering, University of Antwerp, 2000 Antwerp, Belgium; 7 Department of Physiology and Systematics of Micromycetes, Zabolotny Institute of Microbiology and Virology, National Academy of Sciences of Ukraine, 03143 Kyiv, Ukraine; majka42@ukr.net

**Keywords:** bacterial endophytes, biofilm, *Deschampsia antarctica* Desv., PGPB, plant–microbial interactions, amyloids, *Colobantus quitensis* (Kunth) Bartl

## Abstract

*Deschampsia antarctica* and *Colobantus quitensis* are the only two vascular plants colonized on the Antarctic continent, which is usually exposed to extreme environments. Endophytic bacteria residing within plant tissues can exhibit diverse adaptations that contribute to their ecological success and potential benefits for their plant hosts. This study aimed to characterize 12 endophytic bacterial strains isolated from these plants, focusing on their ecological adaptations and functional roles like plant growth promotion, antifungal activities, tolerance to salt and low-carbon environments, wide temperature range, and biofilm formation. Using 16S rRNA sequencing, we identified several strains, including novel species like Hafnia and Agreia. Many strains exhibited nitrogen-fixing ability, phosphate solubilization, ammonia, and IAA production, potentially benefiting their hosts. Additionally, halotolerance and carbon oligotrophy were also shown by studied bacteria. While some Antarctic bacteria remain strictly psychrophilic, others demonstrate a remarkable ability to tolerate a wider range of temperatures, suggesting that they have acquired mechanisms to cope with fluctuations in environmental temperature and developed adaptations to survive in intermediate hosts like mammals and/or birds. Such adaptations and high plasticity of metabolism of Antarctic endophytic bacteria provide a foundation for research and development of new promising products or mechanisms for use in agriculture and technology.

## 1. Introduction

While the most commonly found terrestrial photosynthetic organisms in the Antarctic region are lichens (around 380 species) and bryophytes (130 species) [1], *Deschampsia antarctica* Desv. and *Colobanthus quitensis* (Kunth) Bartl. are the only Magnoliophyta colonized in the Antarctic region. *D. antarctica* (or Antarctic hairgrass) belongs to the Poaceae family, while *C. quitensis* (or Antarctic pearlwort) is a member of the Caryophyllaceae. The reason for their sole presence in Antarctica is still debated as there is no understanding on how only two unrelated flowering plants managed to establish breeding populations in this part of the world with such challenging conditions [2]. A decade ago, climatic and ecological studies concluded that the success of these two species was not primarily due to their unique adaptations, but rather to their long history of migration and adaptation to the region [3].

However, multiple in vitro studies have shown a great positive correlation between bacteria presence and activity and plant physiology adaptations, such as improvement in salt [4,5] and cold tolerance [6,7,8]. Plants host a diverse community of microorganisms that play a critical role in their growth, survival, and establishment by enhancing their resistance to abiotic stress, enabling them to grow in challenging environments [9] through various mechanisms, which are not fully studied yet. Plants present three types of environments conducive for the microbiota: (1) the rhizosphere, where microorganisms interact with the soil and roots, which also contains many exudates from the plant; (2) the inside environment in the tissues of the plant (endosphere); and (3) the phyllosphere, which comprises the surface of the leaves and stems [10]. The role of endophytic bacteria in plant–microbial interactions can vary from pathogenic to beneficial, including mutualistic, commensal, or neutral [11]. Pathogenic or mutualistic behavior may depend on various factors including the plant genotype, the environment, and the co-colonizing microbiota [12]. Microorganisms involved in beneficial association with the plant [13] are frequently called plant growth-promoting bacteria (PGPB) [14,15,16]. They promote plant growth and development by providing nutrients and minerals, such as iron, nitrogen, and phosphorus; synthesizing phytohormones [15]; defending plants from fungal infections acting as biocontrollers [17]; and eliciting plant resilience to biotic and abiotic stress [13]. Endophytes also have a direct contribution to growth promotion by the secretion of such molecules as indole-3-acetic acid (IAA) and affect plant physiology by plant growth regulators such as cytokinins [18]. On the other hand, they are engaged in plant stress responses mainly by reducing the inner-plant stress hormone ethylene levels through ACC deaminase-mediated hydrolysis [6]. Endophytes play a significant role in plant growth promotion by providing fixed nitrogen, a crucial nutrient that is often limited in soil environments. In return, the microorganisms benefit from the bioproducts exuded by the plant root, such as the release of various carbon sources generated by photosynthesis [19].

While research exploring the role of planktonic PGPB in plant growth and stress tolerance has flourished, a crucial aspect remains relatively overlooked: the significance of biofilm formation by PGPB for their survival, colonization, and ultimately, their ability to benefit plants [20]. In the face of harsh environmental challenges, biofilm formation emerges as a crucial strategy for PGPB to thrive in the complex and dynamic plant environment. Biofilm formation equips PGPB with the resilience and adaptability necessary to persist and colonize this intricate environment. This protective shield not only safeguards the bacteria themselves but also enhances their survival in extreme conditions, ultimately benefiting the growth and productivity of their host plants [21].

Numerous studies have shown that the composition of endophytic communities can have a significant impact on plant phenotypic traits. To fully comprehend the selective forces that shape endophytic communities and how these communities adapt to the plant environment and temperature changes, further research is needed. This knowledge could open up new avenues for optimizing plant productivity and performance by manipulating the bacterial constituents of endophytic communities [13]. Here, we investigate possible mechanisms by which bacteria associated with Antarctic vascular plants may have adapted to an endophytic existence.

## 2. Materials and Methods

The materials, plant samples, were collected during the 25th Ukrainian Antarctic Expedition (January–April 2020) along the western part of the Antarctic Peninsula (WAP) (Table 1).

**Figure 1:**
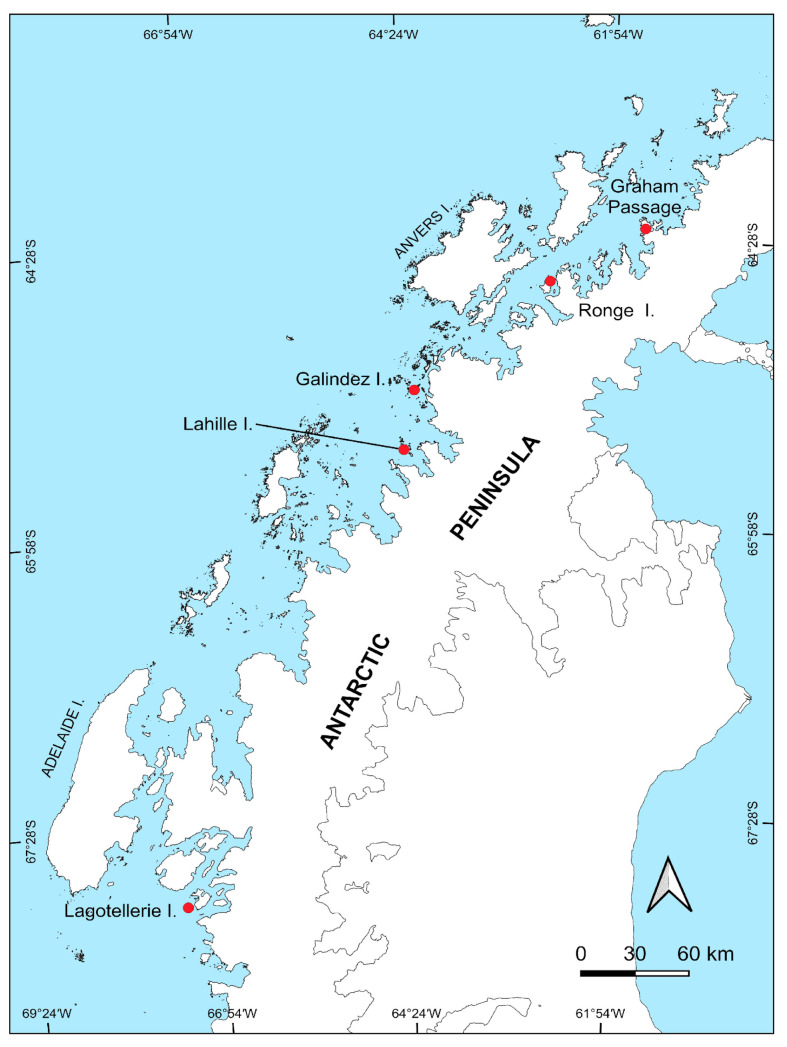
Map providing specific points where plant samples were collected during the 25th Ukrainian Antarctic Expedition (January–April 2020) along the western part of the Antarctic Peninsula (WAP).

Alive plants were collected in the central part (Lahille Is., Ronge Is., Santos Peak in Graham Passage, Galindez Is.) and southern parts of WAP (Lagotellerie Is.), which are presented in Figure 1. The coordinates of the sampling locations are presented in Table 1. Samples of *D. antarctica* included a part of the whole part (both roots and stems and leaves), while samples of *C. quitensis* included the aboveground part only. Alive plants were collected by sterile forceps in sterile plastic containers and transported to the Ukrainian Antarctic Akademik Vernadsky station for further processing. Plant surface sterilization and bacteria isolation were immediately performed at the Akademik Vernadsky station.

Species were identified according to their morphological parameters. In particular, *D. antarctica* (Poaceae) forms tight tussocks. The plants most often form low, dense tufts. According to Barcikowski et al. [22], short culms of *D. antarctica* are thin, straight, and smooth. Leaf sheaths are smooth, connate together, and are straw-like on vegetative shoots, with prominent rough veins on the adaxial side. Ligule is up to 1.5 mm long. Inflorescence shoots (if present) are less numerous but longer than vegetative shoots. The panicles possess characteristic curving in the basal part of the inflorescence axis. Spikelets are large (about 6 mm long) and usually two-flowered. The callus has short hairs. Mature flowers usually remain closed, closely wrapped with glumes. *C. quitensis* (Caryophyllaceae) is a low herbaceous cushion plant of a 1.5 ± 5 cm height, with sessile, linear to linear–triangular leaves; an acute apex; and a base forming a colorless sheath [23].

To isolate endophytic bacteria, the surface sterilization of the plants was performed according to [24] with modifications. Several steps of sterilizations were prolonged to ensure the sterilization of the plants’ surface [25,26]. In particular, the following steps were carried out with plant material: (i) washing in tap water to get rid of crude soil particles; (ii) vortexing in sterile distilled water for 3 min (2 times); (iii) vortexing in EtOH 70% for 2 min; (iv) exposure in NaClO_4_ 5.6% for 10 min; (v) vortexing in EtOH 70% for 2 min; (vi) vortexing in sterile distilled water for 3 min (3 times). Water from the last washing step was inoculated on a nutrient agar medium (CASO, Merck, San Jose, CA, USA) to test the sterility of the washed plant material.

After washing, the plant material was crushed in a sterile mortar with a pestle, and homogenized biomass was diluted and resuspended in 0.9 mL sterile NaCl (0.9%) and used for tenfold dilutions of the plant biomass. In total, 0.1 mL of each dilution was inoculated on solid CASO media (Merck, San Jose, CA, USA) amended with a 1% ethanol extract of *D. antarctica* and 1% methanol. Bacteria were cultured at +18 °C for a week. Bacteria with a distinct colony morphology were inoculated repeatedly on the same nutrient media to receive pure isolates of the endophytic bacteria. Morphotypes of the colonies were described. Bacteria were stained with Gram Stain Kit (Difco, Atlanta, GA, USA) and viewed at 1000× (Konus Academy microscope, Verona, Italy).

For this study, 12 bacterial cultures isolated from *D. antarctica* and *C. quitensis* samples were investigated [25]. These particular cultures were chosen among over 130 isolates based on preliminary studies of their potential benefit role for plants. The source of their isolation is presented in Table 1.

DNA was extracted from overnight cultures of bacteria grown in a CASO (Merk, San Jose, CA, USA) liquid medium with HigherPurity™ Bacterial DNA Isolation Kit (Canvax, Valladolid, Spain) according to the manufacturer’s instructions. Before extraction, bacteria cells were washed in a PBS buffer and pelleted by centrifugation.

16S rRNA genes were amplified in DNA of bacterial cultures (9.1, 10.1, 10.4, 23.2, 24.4, 25.2, 26.2), set 27F (5′-AGAGTTTGATCCTGGCTCAG-3′)/1492R (5′-GGTTACCTTGTTACGACTT-3′). The PCR amplification products were separated using electrophoresis on a 1% agarose gel and then visualized in a UV transilluminator (UVP GelDoc-It 310 model, Ultra-Violet Products Ltd., Cambridge, UK). PCR products were purified using the GeneJet Gel Extraction Kit (Thermo Fisher Scientific Baltics, Vilnius, Lithuania) according to the manufacturer’s instructions and sent to a sequencing service (Macrogen, Amsterdam, The Netherlands).

The 16S rRNA genes’ sequences of bacterial cultures 15.6, 16.7, 26.7, and 40.1 were retrieved from the whole-genome sequencing data. Sequences with an average length of 150 bp were obtained by sequencing on Illumina Novaseq 6000, quality-checked with FastQC 0.11.8 [27], and assembled with Unicycler 0.5.0 [28]. barrnap 0.9 [29] was used to retrieve 16S rRNA sequences from the assembled data.

Sequences were uploaded to the National Center for Biotechnology Information (NCBI) database under accession numbers PP087390, PP087386, PP087387, PP087388, PP087389, PP087357, PP087358, PP087359, PP087360, PP087361, PP087362, and PP087363.

The sequences were compared to similar sequences and their results published in GenBank using BLAST in the NCBI (National Center for Biotechnology Information) database (http://www.ncbi.nlm.nih.gov/blast, accessed on 7 January 2024). It was found that some bacterial sequences have a homology percentage <97% with the closest homologs or have similarity simultaneously for several homologs, which makes it impossible to identify bacteria up to the species level.

The presence of saccharolytic enzymes in bacterial isolates was detected using Hugh–Leifson’s “His’s Streak” (HLS) media. The isolates were inoculated into the HLS media, which contained a variety of carbon sources, including glucose, fructose, mannose, galactose, arabinose, xylose, ribose, lactose, sucrose, and maltose. The presence of saccharolytic enzymes was determined by the color change in the bromothymol blue indicator in the media. Bacteria were cultivated at 26 °C and the color changes were checked at 24, 48, and 72 h after inoculation.

The ability of microorganisms to fix molecular nitrogen as the sole source of nitrogen was detected on a nitrogen-free medium (NF) supplemented with sucrose. Bacteria were cultivated at 26 °C and 140 rpm in a shaking incubator during 96 h. The presence of bacterial growth was identified by a change in the medium OD (λ = 600 nm, UV-Vis spectrophotometer Ulab, Shanghai, China) and as the CFU number on NF agarized media by the sector inoculating method (according to Gold-Rodoman).

A drop collapse assay was performed for assessing biosurfactant (BSF) production according to [30] using a parafilm hydrophobic film. Reduction in the surface tension and collapse of the droplet (consisting of 10 μL aliquots of bacterial overnight culture) indicated the presence of biosurfactants.

A motility assay was performed in tubes filled with semi-solid nutrient agar (0.5%, 10 mL). Bacterial overnight cultures were inoculated by the stab culture method. Cell distribution was measured at 24, 48, and 72 h after inoculation.

Exoprotease production was tested using a skim milk agar [31]. A cleared zone surrounding bacterial growth after incubation for 48 and 72 h at 26 °C was evidence of exoprotease production.

Phosphate solubilizing ability was tested on a Pikovskaya (PVK) medium with Ca_3_(PO_4_)_2_ incorporated [32]. Isolates were incubated at 26 °C for 48 h. A clear zone around the colony indicates phosphate solubilization by the isolated strains. The phosphate solubilization index (PSI) was calculated as the ratio of the total diameter (colony + halo zone) to the colony diameter [33]. All the observations were recorded in triplicate. Strains developing clear zones around their colonies could easily be identified as PSBs.

Bacterial isolates for IAA production were inoculated in NB supplemented with 0.5% (*w*/*v*) L-tryptophan and incubated at 26 °C for 72 h. Liquid cultures were centrifuged at 4000× *g* rpm for 10 min and a supernatant was collected in a fresh sterile tube. For the detection of IAA production, 1 mL of the supernatant was mixed with 2 mL of Salkowski’s reagent and incubated in darkness for 30 min. The production of IAA was indicated by the change in the color of the mixture to pink. The absorbance (OD) of the resulting pigment was read at 535 nm using a UV-Vis spectrophotometer (Ulab, Shanghai, China), and the concentration was calculated using a calibration curve prepared with different concentrations of analytical-grade commercially procured indole-3-acetic acid (IAA). The IAA concentration was then recalculated considering the biomass increase in each culture and normalized to values of 1.0 for OD600 nm on the spectrophotometer.

Hydrogen cyanide production was monitored by using cyanide detection paper placed on Petri dish lids. Single isolates were streaked onto TSA supplemented with 4.4 g glycine L-1 to screen for cyanide production and pH adjusted to 7.4. This medium stimulates the production of HCN, thus enabling the determination of the maximum potential for HCN production. Thereafter, the Petri dishes were inverted. A piece of filter paper impregnated with 0.5% picric acid and 2.0% sodium carbonate was placed in the lid of each Petri dish. The Petri dishes were sealed with parafilm and held at 20 °C for 72 days.

The evaluation of ammonia production was conducted by inoculating bacterial cultures overnight in 10 mL of sterile peptone water and incubating at 26 °C for 48 h and 140 rpm on a shaking platform. After incubation, 0.5 mL of Nessler’s reagent was added to each culture. The development of slight yellow to brownish color was an indicator of ammonia production.

All bacterial cultures were tested separately in NB tubes supplemented with NaCl concentrations ranging from 1, 3, 6, 8, 10, 15, 20, to 25% for salt tolerance. These tubes were inoculated with 0.1 mL overnight cultures. A tube without inoculation served as the negative control for each range of salt. The isolates were incubated at 26 °C for 72 h. The strain that could grow at a particular range of salt was considered as tolerant by observing the presence or absence of growth and comparing it with the negative control, respectively, for each isolate. The growth rate was evaluated by the optical density change at a 600 nm wavelength using a UV-Vis spectrophotometer (Ulab, Shanghai, China). Maximum tolerated concentration (MTC, mg NaCl/L) is described as the highest concentration of NaCl that allows a strain to grow. Along with MTC, we have used the growth reduction rate (GRR, %) to describe the effect of MTC on bacterial growth. The growth reduction rate was calculated according to the formula 100 − (ODstrain − ODcontrol)/100. There are also OD strain − OD_600_ at a particular concentration of NaCl and ODcontrol − OD_600_ in a regular medium without an additional concentration of NaCl.

The ability to grow in oligotrophic environments was tested much similarly to the NaCl test. However, bacteria were cultivated in whole nutrient media (Nutrient Broth, HiMedia Ltd., Maharashtra, India). In order to mitigate oligotrophic environments, the basic media were diluted 2 and 10 times with sterile saline and represented 300 and 60 mg C/L correspondently. The carbon concentration was calculated based on earlier studies [34].

Antifungal activity of bacterial isolates was tested with the agar disk-diffusion method [35]. Six phytopathogenic fungi cultures were obtained from the national collection of D.K. Zabolotny Institute of Microbiology and Virology of the National Academy of Sciences of Ukraine (Department of Physiology and Systematics of Micromycetes), *Nigrospora oryzae* 15966, *Fusarium solani* 50718, *Nectria inventa* 3041, *Botrytis cinerea* 16884, *Sclerotinia sclerotirum* 16883, and *Rhizoctonia solani* 16036. A 5-day mycelium of a pregrown fungi culture was placed in the middle of the Petri dish with a sterile needle. Overnight bacterial cultures were inoculated equidistantly from the fungus and incubated for five days at 26 °C. The results were presented as a percentage of fungal growth inhibition.

Bacteria biofilm formation was studied in the wide temperature range, cultivating bacteria at 4, 26, 37, and 42 °C for 72 h in microcosms with 5 mL of the NB medium. Bacterial growth was detected by measuring absorption at OD600 using a UV-Vis spectrophotometer (Ulab, Shanghai, China). The cell attachment level was measured using the crystal violet assay [36].

Bacteria were cultured aerobically at 26 °C and liquid cultures were cultured in the NB medium, shaking to provide inocula. Overnight cultures of all the strains were used to produce biofilms in stationary NB media microcosms containing 5 mL each. All biofilms were obtained at 26 °C and harvested in three days. Air–liquid–surface biofilms formed on the top of media and solid–liquid biofilms formed onto a bottom surface of a microcosm and were sampled and put on microscopic glass slides for the following staining with 20 μg/mL propidium iodide, 1 mM AmyGreen, and 5 µg/mL of Calcofluor White (CW).

A Confocal Laser Scanning Microscopy (CLSM) analysis of the samples was performed using a Leica TCS SPE Confocal system with a coded DMi8 inverted microscope (Leica, Wetzlar, Germany) and Leica Application Suite X (LAS X) Version 3.4.1 software. Images were obtained with ex/em 537/618 nm for Pi, ex/em 488/510–605 nm for AmyGreen, and ex/em 380/475 nm for CW.

The software MEGA11 (version 11.0.10) was used for a phylogenetic analysis. First, we implemented sequence alignment using MUSCLE algorithms, and then the phylogenetic analysis was performed using the neighbor-joining statistical method and test of phylogeny by the bootstrap method (bootstrap replications—500).

A cluster analysis was performed using the package cluster [37] in R (version 4.3.2, R Core Team, 2023) and RStudio 2023.12.0 Build 369 for Windows.

## 3. Results

### 3.1. Identification and Phylogenetic Analysis

**Figure 2:**
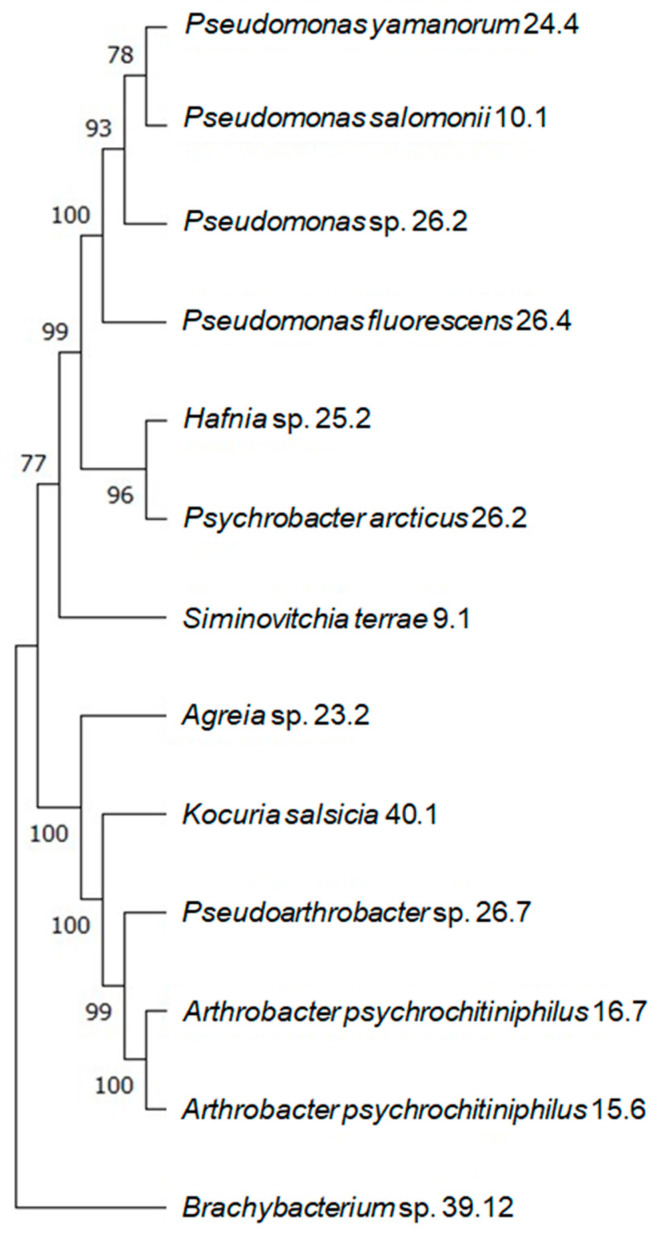
A phylogenetic dendrogram showing the positions of studied strains among each other. The percentage of replicate trees in which the strains were grouped together in a bootstrap test (500 replicates) is shown right near the branches. The shown bootstrap values indicate the confidence that can be placed in the grouping of the strains. The higher the bootstrap value, the more likely it is that the grouping is correct.

A total of 12 different bacterial strains isolated from the Deschampsia and Colobantus samples were obtained from the National Antarctic Scientific Center (Kyiv, Ukraine). *Pseudomonas salomonii* 10.1, *Psychrobacter arcticus* 10.4, and *Brachybacterium* sp. 39.12 were isolated from *C.quitensis*. Based on the 16S sequences obtained from these isolated bacteria, a phylogenetic tree was constructed for all the strains that were isolated (Figure 2).

According to this tree, these strains could be divided into three main groups:

− The Pseudomonas species (24.4, 10.1, 26.2), *Hafnia* sp. 25.2, and *Psychrobacter arcticus* 10.4;
− *Arthrobacter psychrochitiniphilus* 15.6 and 16.7, *Pseudarthrobacter* sp. 26.7, *Kocuria salsicia* 40.1, *Agreia* sp. 23.2, and *Siminovitchia terrae* 9.1;
− *Brachybacterium* sp. 39.12.

Alternatively, a group consisting of *Hafnia* sp. 25.2 and *Psychrobacter arcticus* 10.4 could be considered a separate group as well.

### 3.2. Saccharolytic Enzymes of Bacterial Isolates

The isolates were found to possess a wide range of saccharolytic enzymes for the utilization of mono- and disaccharides (Table 2).

The offered monosaccharides included both hexoses and pentoses, while the disaccharides were represented by lactose and sucrose. The isolates were found to be able to utilize a wide range of sugars, with glucose, mannose, and xylose being the most commonly utilized. Mannose is an isomer of glucose and a component of many polysaccharides and mixed biopolymers of plant, animal, and bacterial origins. As the main component of xylan polymers in plants, xylose is considered one of the most abundant carbohydrates on Earth after glucose. Isolates *Hafnia* sp. 25.2 and *Kocuria salsicia* 40.1 utilized the most sugars out of all the isolates, with eight out of nine, whereas *Siminovitchia terrae* 9.1 utilized the fewest sugars, with only two out of nine (glucose and mannose). The ability of microorganisms to metabolize a wide range of carbon sources, participate in global cycles of transformation of major biogenic elements, and function in harsh environments can be the basis for plant adaptation to adverse environmental factors [38].

### 3.3. Plant Growth-Promoting Traits

To investigate the relationship between the bacteria and the host plant, a number of typical plant growth-promoting traits (PGPTs) were investigated. The potential for nitrogen assimilation from N_2_ was investigated by growing the isolated strains on a nitrogen-free medium (NF) while assessing whether the bacterial strains were able to thrive. Most of the strains showed moderate to strong growth (eight strains; see Table 3) with only one *A. psychrochitiniphilus* strain (16.7) as well as *K. salsicia* being unable to grow at all. Most strains also exhibited the capacity for phosphate solubilization, with only two strains showing a negative result, i.e., the *A. psychrochitiniphilus* strain (16.7) as well as *Agreia* sp. Additionally, only four strains were found to produce any IAA (*Hafnia* sp., *A. psychrochitiniphilus* 15.6 but not 16.7, and *K. salsicia*). Two strains, *Hafnia* sp. 25.2 and *Pseudomonas yamanorum* 24.4, were able to produce HCN. Additionally, all studied strains were evaluated as positive for ammonia production. Lastly, no exoprotease activity was detected in any of the studied strains under tested conditions.

### 3.4. Salt and Oligotrophic Environment Tolerance

According to the results, endophytic isolates could be described as halotolerant bacteria. Thus, among studied bacteria, two strains (*Pseudarthrobacter* sp. 26.7 and *Kocuria salsicia* 40.1) were characterized with the highest salt tolerance and were able to tolerate 25% and 20% of NaCl in the media correspondently. Half of the rest of the studied bacteria were able to tolerate 10% of NaCl, whereas *Agreia* sp. 23.2, *Hafnia* sp. 25.2, and *Pseudomonas* sp. 26.2 tolerated 6%, and *Pseudomonas salomonii* 10.1 and *Pseudomonas yamanorum* 24.4 tolerated 3% (Table 4).

**Figure 3:**
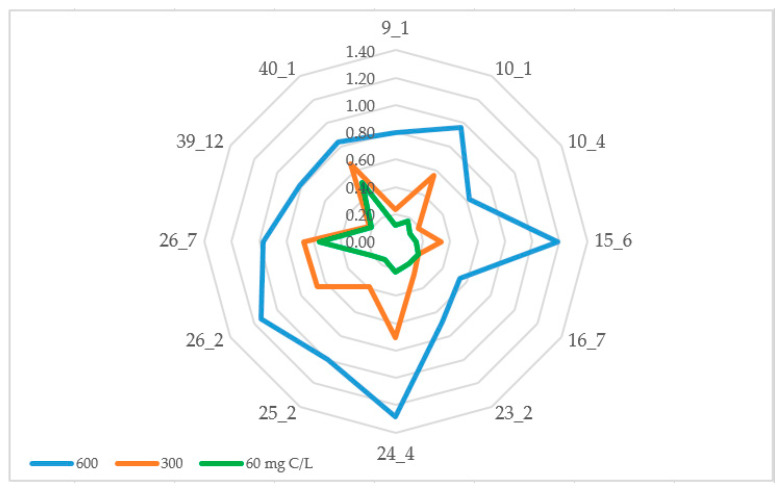
Growth rate (OD_600_) of endophytic bacteria in oligotrophic environments.

Interestingly, those two strains highly resistant to NaCl were also quite adaptive to carbon oligotrophic environments (Figure 3).

Their growth reduction rates were 42.31% and 40.57% correspondently, when they were grown in 1/10 diluted media (60 mg C/L) compared to the whole media (600 mg C/L). Based on this parameter, we can also describe *A. psychrochitiniphilus* 16.7 as an oligotrophic isolate (GRR = 65.01%). The GRR of other studied bacteria varied between 72.42% (*Agreia* sp. 23.2) and 84.91% (*S. terrae* 9.1). Taking into account their growth rate, not dropping lower than 0, 12 OD_600_, we can describe those bacteria as oligotolerant as well.

### 3.5. Antifungal Activity

**Figure 4:**
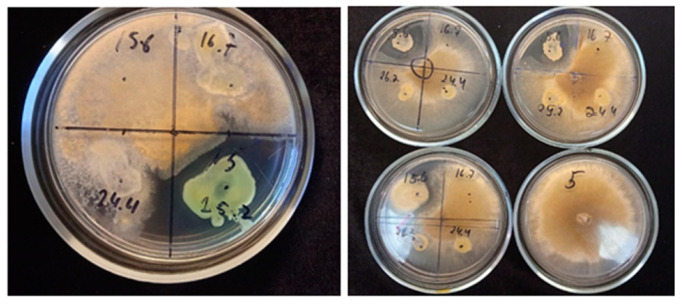
Examples of fungal growth inhibition of *Botrytis cinerea* 16884 by studied bacteria *Hafnia* sp. 25.2. and *A. psychrochitiniphilus* 15.6. Growth of fungi on 5th day of cultivation, 26 °C [39].

Among the isolated bacterial strains, only three (*Arthrobacter psychrochitiniphilus* 15.6, *Pseudomonas yamanorum* 24.4, and *Hafnia* sp. 25.2) showed antifungal activity against phytopathogenic fungi (Table 5; Figure 4). The other strains had no effect on the growth of the selected fungal strains.

### 3.6. Biofilm Growth and Structure

**Figure 5:**
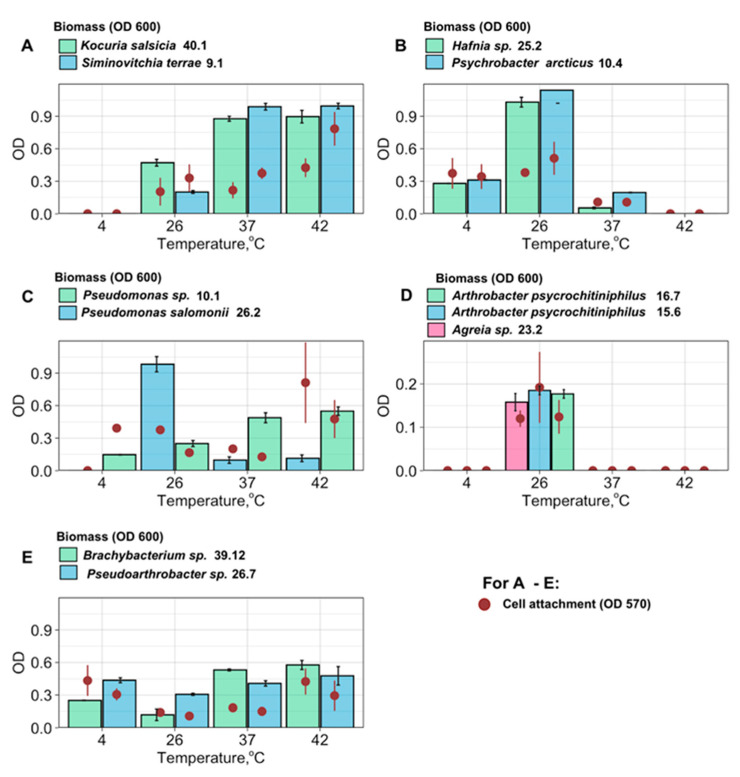
Biofilm formation of Antarctic endophytic bacteria in wide temperature range. (**A**–**E**): different types of temperature-dependent behavior.

Both biomass production and cell attachment were measured for 12 bacterial strains (Figure 5). In general, both biomass production and attachment followed one of five similar patterns, which could be characterized using five distinct graph types, each based on the levels of biomass production and cell attachment observed across the tested temperature range (Figure 5, groups A–E). Group A (consisting of *S. terrae* and *K. salsicia*) did not show any growth at all at 4 °C, and the highest growth rates at 37–42 °C (correlating with the strongest attachment). Group B (consisting of *P. arcticus* and *Hafnia* sp.) has a broad temperature optimum with high growth rates and strong attachment around 26 °C and slower growth and less strong attachment at 4 °C and 37 °C. Group C (*Pseudomonas* sp. and *P. salomonii*) exhibited the strongest biofilm growth at 26 °C and the strongest attachment around 42 °C. Group D comprises the bacteria that formed a biofilm only at 26 °C (*A. psychrochitiniphilus* and *Agreia* sp.), and Group E are bacteria that grow at all temperatures, but with a minimum at intermediate temperatures, and maximal attachment at 4 °C and 37–42 °C (*Pseudarthrobacter* sp. 26.7, *Brachybacterium* sp.).

**Figure 6:**
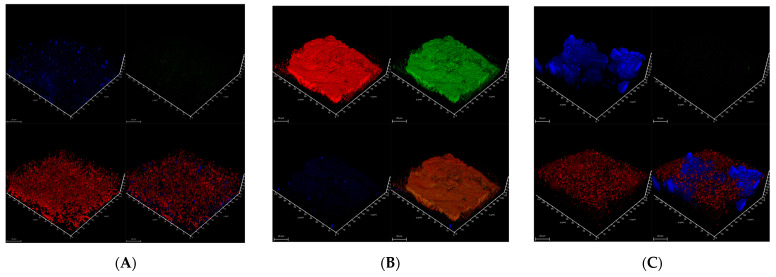
CLSM imaging of 3-day-old single-species bacterial biofilms. Calcofluor White (blue channel) was used to visualize cellulose, AmyGreen (green channel) was used to visualize amyloid proteins, propidium iodide (red channel) was used to visualize eDNA, and, respectively, all three channels are combined in the bottom-right image. The scale bars indicate 20 µm. *(***A**)—*Siminovichia terrae* 9.1.; (**B**)—*Pseudomonas salomonii* 10.1; (**C**)—*Arthrobacter psychrochitiniphilus* 15.6.

The structure of the biofilms could be visualized by CLSM, which allowed us to classify the different biofilm as dense (i.e., 10.4, 40.1), mucous (i.e., 15.6, 16.7, 26.2), and dispersed (i.e., 9.1, 26.7) structural types (as described in Table 4). In each of these biofilms, three essential components (described in detail in [38]) were assessed: bacterial cellulose (with Calcofluor White), amyloid proteins (with AmyGreen), and extracellular DNA (eDNA, with propidium iodide). Typical examples of these structures of bacterial biofilm types are presented in Figure 6A–C:

− *Siminovitchia terrae* 9.1 (Figure 6A) forms a thick aggregate (on average, 32 µm thick) of cellulose fibers and cell clusters strongly intertwined. The biofilm profile was dispersed, and only low amyloid contents were found in this biofilm.
− *Pseudomonas salomonii* 10.1 (Figure 6B) forms a dense biofilm with high levels of amyloid and eDNA, and relatively low cellulose content.
− An example of a mucous biofilm profile is biofilm formed by *Arthrobacter psychrochitiniphilus* 15.6 (Figure 6C) with high cellulose and eDNA content, and almost no amyloids. The cellulose aggregates into ball-shaped structures penetrating the whole biofilm but not intertwined with cells as was observed in *P. salomonii* (Figure 6B).

## 4. Discussion

The Antarctic continent is characterized by its harsh environmental conditions, and hosts a limited array of flora, with *D. antarctica* and *C. quitensis* being the only vascular plants to have successfully colonized this extreme landscape. Despite the seemingly inhospitable nature of their habitat, these plants thrive, owing in part to the plant promoting capacities of the associated microbiota. This study explores capacities of bacterial biofilm-formers isolated from the endosphere of the Antarctic vascular plants collected in the central and southern regions of the western side of the Antarctic Peninsula (WAP). Our analysis centers on the phylogeny, plant growth-promoting traits, salt tolerance, and biofilm structure and physiology of the newly isolated bacterial cultures.

### 4.1. Novel Species

The habitat of the vascular plants and the plant communities they create is largely defined by the climate of the region. Mean temperatures along a north–south transect on the WAP fluctuate between −3 °C and −10 °C [40]. In the period of 1996–2026, the minimum temperature recorded on the Argentina Islands (central part of WAP) has been −28.6 °C, and the maximum has been +8.2 °C. The average summer temperature has been 0.7 °C with a dispersion of 1.8, but the amplitude has reached 10 °C [41].

WAP exhibits a climatic latitudinal gradient, with climate severity increasing from north to south [42]. This increase in climatic severity is evidenced by a shorter growing season, lower temperatures, and reduced precipitation, along with heightened aridity, continentality, and sunshine towards the south. The latitudinal lapse rate for the west coast region is calculated at −0.77 °C per degree latitude [43].

Few studies have been performed so far on the presence of endophytic bacteria on the Antarctic continent. Zhang et al. [44] already identified endophytes of the genera *Pseudomonas*, *Bacillus*, and *Micrococcus* in *D. antarctica*; using high-throughput DNA sequencing [45], they found a wide array of Pseudomonadaceae, Enterobacteriaceae, and Microbacteriaceae, in the endosphere of both *Deschampsia antarctica* and *Colobanthus quitensis.* Lee et al. [46] found *Acenetobacter johnsonii*, *Acidovorax radicis*, *Fluviicola taffensis*, *Pectobacterium carotovorum*, *Frigoribacterium faeni*, and several *Pseudomonas* and *Flavobacterium* species in the Antarctic moss *Sanionia uncinata*. Even Antarctic lichens offer a wide range of endobacterial species, belonging to the Alphaproteobacteria, Actinobacteria, Betaproteobacteria, Gammaproteobacteria, Bacteroidetes, Firmicutes, and Deinococcus-Thermus [47]. With the present study, new genera and species have been identified as possible inhabitants of the endosphere of *D. antarctica* and *C. quitensis*, i.e., *Hafnia* sp., *Psychrobacter arcticus* (belonging to the Hafniaceae and Moraxellaceae families of the Pseudomonadota phylum, Gammaproteobacteria class), *Agreia* sp., *Kocuria salsicia*, *Pseudarthrobacter* sp., *Arthrobacter psychrochitiniphilus*, and *Brachybacterium* sp. (*Microbacteriaceae*, *Micrococcaceae*, and *Dermabacteraceae* families of the *Micrococcales* order, *Actinomycetes* class).

### 4.2. Cluster Analysis

**Figure 7:**
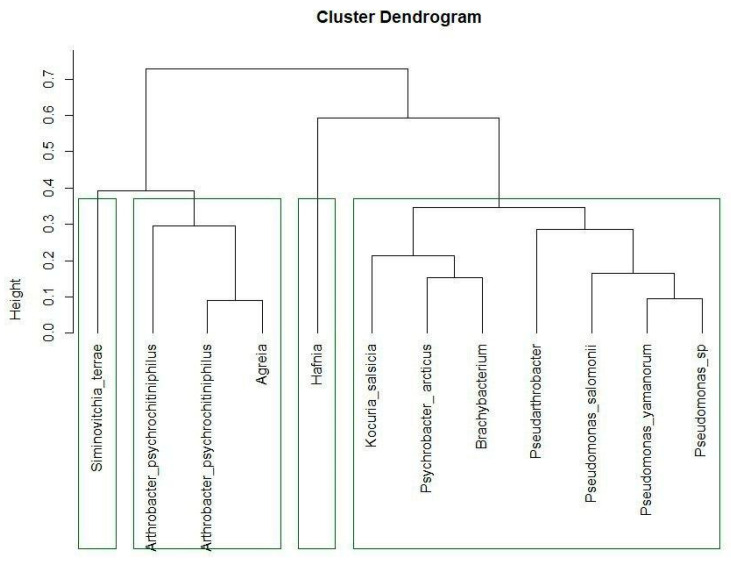
The cluster analysis of the different bacterial strains based on a Euclidean distance matrix.

To understand the behavior of these bacterial strains, different groups of bacteria were identified on a functional basis (Figure 7). The first cluster consists of *S. terrae*, *A. psychrochitiniphilus* (15.6 and 16.7), and *Agreia* sp. They are all psychrotolerant bacteria with the potential to play important roles in the ecology of cold environments. They are all capable of degrading chitin [48], which could be important for cycling nutrients in these environments. These strains also create similar biofilms, consisting of cell aggregates embedded in cellulose fibers, while lacking amyloids. As a side remark, it should be noted that even though strains 15.6 and 16.7 belonged to the same bacterial species—*A. psychrochitiniphilus*—they showed significant differences in their growth-promoting traits.

The second branch consists of only the *Hafnia* strain. Given that this genus was also alternatively separated from others in the phylogenetic tree (Figure 2), this does not come as a surprise. *Hafnia* is also the strain that produces the most IAA (Table 2) and is only one of the three strains identified here that possess antifungal activity (Table 3), which sets it apart from the other groups in terms of its behavior as an endophyte.

The third group harbors first of all the different *Pseudomonas* strains, which are obviously also taxonomically close. They are joined by the strains of *Pseudarthrobacter*, *Brachybacterium*, *Psychrobacter*, and *Kocuria* species. These are Gram-negative rod-shaped bacteria, heterotrophic, and aerobic. All these bacteria formed different types of biofilms with very low amyloid contents. On the other hand, these bacteria show differences in their responses to different temperatures. The *Pseudomonas* strains grow at 4 °C, and have a biofilm growth optimum at 26 °C (Figure 5, group C). *Pseudarthrobacter*, *Brachybacterium*, and *Psychrobacter* are psychrotolerant (Figure 5, group E), while *Kocuria* is not.

### 4.3. Plant Growth-Promoting Properties of Antarctic Bacteria

Endophytic bacteria can directly promote plant growth, typically by improving plant nutrition, or indirectly, by reducing the inhibitory effects of various pathogenic agents on plant growth and development [49]. The positive influence of many endophytic bacteria on plants is mediated by a number of mechanisms, such as improved mineral nutrition (phosphorus and nitrogen) [50], plant growth hormone production (such as auxins, cytokinins, and gibberellins) or the modulation of ethylene levels in plants [51,52] increasing resistance to biotic and abiotic stress [53], the modification of root development [54], and biocontrol [55].

There is a wealth of data on the diversity of the physiological effects caused by beneficial PGPB on various plant organisms, which can be divided into growth-stimulating and protective roles from a wide range of adverse effects [56].

Growth stimulation can be the result of nitrogen fixation, phosphate solubilization, iron sequestration, and synthesis of phytohormones, which helps plants to cope with stress and maintain cell metabolism. Common characteristics among most of the strains studied here were the ability to grow on an NF medium, phosphate solubilization, and the synthesis of biosurfactants. These traits play an important role in plant colonization and promoting plant growth in harsh conditions. The different bacteria identified here all demonstrate at least one of these properties, although the bacteria investigated here show complementary traits suggesting equally complementary ecological relations with the host plants.

Firstly, most bacteria were able to use atmospheric N_2_ as a source of nitrogen (at least for their own growth) and may be able to provide the host plant with an extra source as well (Table 2). After all, nitrogen fixation by endophytic bacteria is a crucial process for plant growth in nutrient-limited environments, such as the Antarctic region [57]. Additionally, all studied bacteria were able to produce ammonia, which could also be used as a nitrogen supplement for plant growth. In addition, bacteria can enhance plant nutrient uptake by solubilizing immobilized phosphates [58]. Again, most of the endophytic bacteria identified here were able to dissolve immobilized mineral phosphates, suggesting that during initial colonization, bacteria can increase the availability of phosphates to plants. Less frequent was the trait to produce IAA, which was observed only in four strains, and HCN production was also observed—only in two. Exoproteases from PGPB play a multifaceted role in plant growth promotion. They contribute to improved nutrient acquisition, root development, and plant defense, and potentially influence plant physiology [59]. However, there was no evidence of exoprotease activity in the strains under the tested conditions. Our results suggest that the endophytic bacteria might not rely on exoprotease activity for nutrient acquisition or other relevant functions within the plant host. Endophytic bacteria may have evolved alternative strategies for nutrient acquisition or interaction with the plant host that do not require exoprotease secretion.

Other traits indicated the possibility that at least some of the bacterial strains could help the host plant defend itself from fungal infections. Firstly, the ability to synthesize BSF was demonstrated by almost all isolates. Although BSFs are known as biocontrol molecules, their role is probably more complex [60] and may also allow for further interaction with biofilms [61], and as such may affect not only pathogens, but also regulate colonization activity and the balance between the endophytic microbial community itself. Alternatively, *A. psychrochitiniphilus*, *P. yamanorum*, and *Hafnia* sp. were able to block the growth of several common phytopathogenic fungi (Table 3). *Arthrobacter* sp. and *Pseudomonas* sp. have demonstrated antifungal activity before [62] and are of potential use as biocontrol agents in agricultural studies [63]. Mechanisms supporting antifungal activity may include syntheses of volatile organic compounds such as dimethylhexadecylamine [63], or the presence of biosurfactants in the form of cyclic lipopeptides [64], but whether these mechanisms apply here should be a question for future research.

Surprisingly, not all of the studied isolates were psychrotolerant (Figure 5, group A, C, and D), which suggests that these bacteria only form biofilms during periods of warmer weather. Other species (Figure 5, group E) have a wide cultivation temperature range, and have higher biofilm attachment rates both at colder temperatures (4 °C) and at the higher end (37–42 °C), which suggests that they have a complex evolutionary history and may have an intermediate host (Figure 5). This so-called U-shaped biofilm formation behavior (*Pseudartrobacter* sp. 26.7, *Brachybacterium* sp. 39.12) was described earlier in recent studies of endophytic bacteria [65] as a strategy with increased biofilm formation at both optimal and lower temperatures, suggesting possible cold-adaptation mechanisms. The impact of temperature on biofilm formation of endophytic bacteria is multifaceted and species-dependent. Further research is crucial to fully understand the mechanisms involved and predict how climate change might affect these complex plant–microbe interactions.

As a last remark on how endophytic bacteria may assist in the defense of the host plant against (biotic and abiotic) stress, it should be noted that not every action of an endophytic bacterium is necessarily directly beneficial. It is equally possible that bacteria caused what is known as “eustress”, a low level of stress, which keeps the defense mechanisms of the host plant active and may even promote, indirectly, a healthier metabolism in the long run [66].

### 4.4. Salt Tolerance

According to the growth rate, studied bacteria are not halophilic, but halotolerant microbes instead. There are several categories of halotolerant microbes: non-tolerant, those which tolerate only a small concentration of salt (about 1% *w*/*v*), and slightly tolerant, tolerating up to 6–8% (*w*/*v*) [67]. The majority of studied strains could be characterized as slightly to moderately halotolerant bacteria. Endophytic bacteria residing in Antarctic vascular plants are likely halotolerant (able to tolerate high salt concentrations) due to a combination of evolutionary pressures and the specific characteristics of their environment such as high salinity of Antarctic soils and meltwater [68,69]. Numerous studies have demonstrated that plant-associated bacteria can mitigate plant salt stress through various mechanisms including IAA, ACC deaminase production, and phosphate solubilization, among other chemical compounds [5]. Halotolerance could be beneficial for endophytic bacteria even within plant–microbial interactions, providing a higher survival and fitness rate within the tissues of their host plants.

### 4.5. Biofilm Structure and Physiology

The presence of large amounts of DNA, measured here with the membrane impermeable dye propidium iodide, were due to the presence of extracellular DNA (eDNA), which is an important component of the biofilm matrix [70,71], and a functional absence (or chelation with other compounds) can inhibit the formation of biofilms completely [72]. It can act as an adhesin, and participate in intercellular genetic information transfer for the adaptation of microorganisms and the regulation of microbial–plant interactions [73]. It is therefore logical that eDNA surfaces are in large amounts in all the biofilms produced by the Antarctic endophytes. The second major component in many biofilms consists of amyloid proteins. Amyloids have been reported to play an important role in the promotion of survival and pathogenicity in prokaryotes and a cause of neurodegenerative diseases [74,75]. However, recently, amyloid-based mechanisms have been revealed as crucial players in the complex interactions between microbes within the plant microbiome. Amyloids facilitate microbial adhesion and the formation of a protective coating, shielding microbes from external stressors and fostering cooperation within the community [76]. Amyloids also enable the creation of hydrophobic surface layers, known as hydrophobins, which coat the aerial hyphae and spores of plant pathogenic fungi as well as certain bacterial biofilms [77]. Also, these hydrophobins may contribute to the virulence of any plant pathogens by facilitating dissemination and infectivity. However, apart from some of the *Pseudomonas* strains, none of the bacteria isolated here possess high amounts of amyloids in their biofilms (Table 6). The importance of amyloids should therefore at least be investigated directly in vivo (with experiments already ongoing in our labs), and the role of amyloid proteins in endophytic, commensal relations should not be overestimated.

The results of research reveal possible mechanisms of how bacteria enforce the growth of vascular plants in the Antarctic region. The majority of studied strains adapted to cold environments, being either psychrotolerant (like *Siminovitchia terrae*, *Pseudomonas salomonii*, *Arthrobacter psychrochitiniphilus*, *Pseudarthrobacter* sp.) or psychrophilic (*Psychrobacter arcticus*). Interestingly, half of the strains were able to grow in a wide temperature range and to form biofilms with different characteristics. While some Antarctic bacteria remain strictly psychrophilic, others demonstrate a remarkable ability to tolerate a wider range of temperatures, suggesting that they have acquired mechanisms to cope with fluctuations in environmental temperature and developed adaptations to survive in intermediate hosts like mammals and/or birds. Additionally, such adaptations and high plasticity of metabolism of Antarctic endophytic bacteria provide a foundation for research and development of new promising products or mechanisms for use in agriculture and technology. Many of the strains have unique biochemical capabilities that could be exploited for biotechnological applications. For example, the *Hafnia* genus is a relatively unexplored group of bacteria with the potential for a variety of applications, for example, to produce bacteriocins. This makes them potentially useful in biocontrol applications as well as new pharmaceutical screening.

## 5. Conclusions

The various physiological, plant growth-stimulating, and biofilm forming mechanisms of the bacteria described in this paper demonstrate the power of endophytic bacteria to maintain plant development in cold regions. The ability to grow in the wide temperature range could not only indicate the presence of an intermediate host but also shifts the perception of Antarctic bacteria as those that are strictly evolved to survive in harsh environments of that region and helps to consider these bacteria as potentially useful in agricultural and biopharmaceutical studies.

## Figures and Tables

**Table 1:** List and description of samples.

Number of Sample	Sampling Location	Plant	Isolate Number	Coordinates
1	Lahille Island	*D. antarctica*	9.1	−65.553580°, −64.394883°
2	Lahille Island	*C. quitensis*	10.1, 10.4	−65.553580°, −64.394883°
3	Ronge Island	*D. antarctica*	15.6, 16.7	−64.683430°, −62.644170°
4	Santos Peak, Graham Passage	*D. antarctica*	23.2, 24.4	−64.405750°, −61.547410°
5	Galindez Island, Argentine Islands	*D. antarctica*	25.2, 26.2, 26.7	−65.244807°, −64.255709°
6	Lagotellerie Island	*C. quitensis*	39.12	−67.88486°, −67.38765°
7	Lagotellerie Island	*D. antarctica*	40.1	−67.88486°, −67.38765°

**Table 2:** Saccharolytic enzymes of Antarctic endophytes.

Strain Number	Species Name	Monosaccharides							Disaccharides	
		Hexoses					Pentoses			
		*Glu*	*Fru*	*Man*	*Gal*	*Ara*	*Xyl*	*Ryb*	*Lac*	*Suc*
9.1	*Siminovitchia terrae*	+	-	+	-	-	-	-	-	-
10.1	*Pseudomonas salomonii*	+	-	+	+	-	+	+	-	-
10.4	*Psychrobacter arcticus*	+	-	+	+	-	+	+	-	-
15.6	*Arthrobacter psychrochitiniphilus*	+	-	-	-	-	+	-	-	-
16.7	*Arthrobacter psychrochitiniphilus*	+	-	-	-	+	+	-	+	+
23.2	*Agreia* sp.	+	+	+	-	+	+	-	+	+
24.4	*Pseudomonas yamanorum*	+	-	+	+	-	+	-	-	+
25.2	*Hafnia* sp.	+	+	+	+	-	+	+	+	+
26.2	*Pseudomonas* sp.	+	-	+	+	-	+	-	-	+-
26.7	*Pseudarthrobacter* sp.	+	+	+	-	-	-	+	+-	+
39.12	*Brachybacterium* sp.	+	+	-	-	-	-	+	-	-
40.1	*Kocuria salsicia*	+	+	+	+	+	+	+	+-	+

Note: “+”, positive reaction; “+*-*“, unexpressed positive reaction; “-”, negative reaction.

**Table 3:** Plant growth-promoting traits of Antarctic endophytes.

Strain Number	Species Name	Growth on NF Media		Phosphate Solubilization Index (PSI)	BSFs	Motility	IAA (µg/mL)	HCN
		OD_600_	CFU Number					
9.1	*Siminovitchia terrae*	0.031 ± 0.003	-	1.35 ± 0.02	-	+	-	-
10.1	*Pseudomonas salomonii*	0.052 ± 0.011	10^6^	2.0 ± 0.01	+	+	-	-
10.4	*Psychrobacter arcticus*	0.047 ± 0.006	10^4^	3.23 ± 0.52	+	-	-	-
15.6	*Arthrobacter psychrochitiniphilus*	0.107 ± 0.012	5 × 10^5^	1.53 ± 0.26	-	+	35.7 ± 3.0	-
16.7	*Arthrobacter psychrochitiniphilus*	-	-	-	-	+	-	-
23.2	*Agreia* sp.	0.024 ± 0.001	-	-	-	+	-	-
24.4	*Pseudomonas yamanorum*	0.227 ± 0.029	10^6^	3.11 ± 0.81	+	+	-	+
25.2	*Hafnia* sp.	0.139 ± 0.006	10^8^	3.03 ± 0.28	+	+	544.0 ± 7.0	+
26.2	*Pseudomonas* sp.	0.188 ± 0.005	10^8^	2.71 ± 0.51	+	+	46.1 ± 2.0	-
26.7	*Pseudarthrobacter* sp.	0.225 ± 0.042	10^6^	2.57 ± 0.79	+	-	-	-
39.12	*Brachybacterium* sp.	0.058 ± 0.004	10^3^	2.75 ± 0.34	+	-	-	-
40.1	*Kocuria salsicia*	0.262 ± 0.027	5 × 10^5^	1.55 ± 0.27	+	-	21.3 ± 2.0	-

**Table 4:** Salt tolerance of studied bacteria.

Strain Number	Species Name	MTC, mg NaCl/L	GRR, %
9.1	*Siminovitchia terrae*	10	89.71
10.1	*Pseudomonas salomonii*	3	25.17
10.4	*Psychrobacter arcticus*	10	85.47
15.6	*Arthrobacter psychrochitiniphilus*	10	80.76
16.7	*Arthrobacter psychrochitiniphilus*	10	94.09
23.2	*Agreia* sp.	10	79.40
24.4	*Pseudomonas yamanorum*	3	15.53
25.2	*Hafnia* sp.	6	42.03
26.2	*Pseudomonas* sp.	6	87.25
26.7	*Pseudarthrobacter* sp.	25	77.73
39.12	*Brachybacterium* sp.	10	84.99
40.1	*Kocuria salsicia*	20	55.74

Note: MTC refers to maximum tolerated concentration, mg NaCl/L; GRR—growth reduction rate, %.

**Table 5:** Endophytic bacteria antifungal activity.

Fungal Strain	Inhibition of Fungal Growth, %		
	*Arthrobacter psychrochitiniphilus* 15.6	*Pseudomonas yamanorum* 24.4	*Hafnia* sp. 25.2
*Nigrospora oryzae* 15966	11.13 ± 1.73	-	18.30 ± 1.90
*Fusarium solani* 50718	33.00 ± 8.00	20.33 ± 1.15	-
*Nectria inventa* 3041	-	5.10 ± 0.21	21.85 ± 2.03
*Botrytis cinerea* 16884	18.28 ± 1.58	-	18.90 ± 1.90
*Sclerotinia sclerotirum* 16883	21.61 ± 2.13	11.43 ± 0.57	-
*Rhizoctonia solani* 16036	5.96 ± 0.16	3.37 ± 0.2	20.08 ± 0.21

Note: “-“, zero inhibition.

**Table 6:** Biofilm structure of Antarctic vascular plant endophytes.

Bacterial Strain	Biofilm Type	Average Biofilm Thickness (µm)	Structure Description
*Siminovitchia terrae* 9.1	ALS *	32	Dispersed biofilm; cell aggregates are tightly intertwined with cellulose fibers; very low levels of amyloids
*Pseudomonas* *salomonii* 10.1	ALS	~20	Dense biofilm; low content of cellulose; high content of amyloid proteins
*Psychrobacter arcticus* 10.4	LS **	15	Dense biofilm; low content of cellulose; low content of amyloid proteins
*Arthrobacter* *psychrochitiniphilus* 15.6	LS	45	Mucous biofilm; biofilms are in the form of spherical aggregations; cells are tightly surrounded by cellulose; low amyloids
*Hafnia* sp. 25.2	ALS	5	Dispersed biofilm; high cellulose content; cellulose in fibers intertwined with cells; low amyloid content
*Pseudomonas* sp. 26.2	ALS	35	Mucous biofilm; high content of cellulose and amyloid proteins
*Pseudarthrobacter* sp. 26.7	ALS	20	Mucous biofilm; cell aggregates are very dispersed; cellulose is located between cells, which are surrounded by a thin layer of amyloid proteins
*Brachybacterium* sp. 39.12	LS	7	Dispersed biofilm; low cellulose content; very few amyloid proteins
*Kocuria salsicia* 40.1	LS	25	Dense biofilm, with small dense aggregates of cells with fibers; few amyloid proteins

Note: * ALS is biofilm formed at air–liquid–solid interface, ** LS—biofilm formed at liquid–solid interface.

## Data Availability

The original contributions presented in the study are included in the article, further inquiries can be directed to the corresponding author.
